# Hypereosinophilic syndrome presenting as pleural and pericardial effusion in a Pediatric patient: a rare case report

**DOI:** 10.1093/omcr/omag012

**Published:** 2026-03-23

**Authors:** Sonam Dhenga, Milan Pokhrel, Simran Rauniyar, Bibek Shrestha, Amrit Pandey, Sugam Ale Magar

**Affiliations:** Maharajgunj Medical Campus, Institute of Medicine, Tribhuvan University, Kathmandu, 44600, Nepal; Maharajgunj Medical Campus, Institute of Medicine, Tribhuvan University, Kathmandu, 44600, Nepal; Maharajgunj Medical Campus, Institute of Medicine, Tribhuvan University, Kathmandu, 44600, Nepal; Maharajgunj Medical Campus, Institute of Medicine, Tribhuvan University, Kathmandu, 44600, Nepal; Maharajgunj Medical Campus, Institute of Medicine, Tribhuvan University, Kathmandu, 44600, Nepal; Nepal Medical College, Kathmandu University, Jorpati, Kathmandu, Nepal

**Keywords:** Hypereosinophilic syndrome, pericardial effusion, pleural effusion, Pediatric, case report

## Abstract

Hypereosinophilic syndrome is a rare disorder in children, particularly when presenting with simultaneous pleural and pericardial effusions. We report the case of a 4-year-old girl who presented with fever, cough, shortness of breath, and chest pain. Clinical evaluation revealed bilateral pleural effusion and later pericardial effusion with impending tamponade. Laboratory investigations showed marked hypereosinophilia, while infectious, rheumatologic, and malignant causes were excluded. Bone marrow findings confirmed eosinophilic predominance without atypia. The patient underwent pericardiocentesis and was treated with corticosteroids, resulting in clinical improvement and normalization of eosinophil counts. On follow-up, she remained asymptomatic with resolution of effusions. This case underscores the importance of considering HES as a differential diagnosis in pediatric patients with eosinophilic effusions and highlights the role of early recognition and corticosteroid therapy in preventing life-threatening complications.

## Introduction

Hypereosinophilia is defined as an absolute eosinophil count (AEC) greater than 1500 eosinophils/μl in the peripheral blood on at least two occasions one month apart, and/or tissue hypereosinophilia confirmed by histopathology. Hypereosinophilic syndrome (HES) is characterized by persistent hypereosinophilia accompanied by evidence of end-organ dysfunction, after exclusion of secondary causes [1]. HES is classified into myeloproliferative, lymphocytic, idiopathic, familial, associated, and overlap variants [2].

HES is a rare condition, with an estimated incidence of 0.16–0.36 per 100 000 and a prevalence ranging from 0.36 to 6.3 per 100 000 [3]. The disease can involve multiple organ systems, with dermatological manifestations being the most common, followed by pulmonary, gastrointestinal, cardiac, and neurological involvement [3, 4] Although pulmonary and cardiac involvement may develop during the disease course, eosinophilic pleural effusion [2] and pericardial effusion as initial manifestations are uncommon. Cardiac involvement accounts for most fatalities in patients with HES [5].

HES typically affects individuals between 20 and 50 years of age and shows a marked male predominance, with a male-to-female ratio ranging from 4:1 to 9:1 [6]. Pediatric presentations are rare and often pose a diagnostic challenge due to atypical clinical features. We report a rare pediatric case of hypereosinophilic syndrome presenting with simultaneous pleural and pericardial effusions, highlighting the importance of early recognition and prompt treatment to prevent life-threatening complications.

## Case report

A 4-year-old female with a normal birth and developmental history presented to our center with cough, fever, shortness of breath, and chest pain for 1 week. There was no history of skin rash, wheezing, joint pain, weight loss, night sweats, recent travel, or exposure to tuberculosis. There was no history of prior medication use, including antibiotics, anticonvulsants, or herbal drugs. No known drug or food allergies were reported. Family history was unremarkable, with no history of atopy, autoimmune disease, malignancy, or hypereosinophilic disorders. Her Vital signs revealed a body temperature of 100°F, pulse rate of 110 beats per minute, blood pressure of 90/60 mm of Hg, respiratory rate of 24 breaths per minute, and oxygen saturation of 95% on room air. Respiratory system examination revealed a dull note on percussion of bilateral lung fields, and decreased air entry was present on auscultation of the chest. Abdominal examination revealed normal liver and spleen size.

With this history and examination, pleural effusion was suspected, and investigations were conducted to determine the cause. A complete blood count was done, which revealed a total leukocyte count of 15 000/mm^3^ with eosinophils comprising 41% of total cells (AEC = 6150/mm^3^), Hb 11.3 g/dl, and platelet count of 4,00000. Stool RE/ME, Vit B12 was also evaluated, revealing normal results. Pleural fluid analysis revealed an exudative effusion with a total leukocyte count of 1200 cells/mm^3^, predominantly eosinophils (48%), with lymphocytes (32%) and neutrophils (20%). Pleural fluid protein was 4.2 g/dl, glucose 82 mg/dl, and LDH 210 U/l. Cytological examination showed no malignant cells. Gram stain, Ziehl–Neelsen stain, and bacterial cultures were negative, effectively ruling out bacterial and tubercular infections. Pericardial fluid analysis demonstrated straw-colored fluid with a total leukocyte count of 950 cells/mm^3^, showing marked eosinophil predominance (45%), along with lymphocytes (35%) and neutrophils (20%). Pericardial fluid adenosine deaminase (ADA) was 18 U/l, and LDH was 195 U/l, both within normal limits. Cytological examination was negative for malignant cells. GeneXpert MTB/RIF assay and acid-fast bacilli staining of the pericardial fluid were negative, thereby excluding tubercular and malignant etiologies.

**Figure 1:**
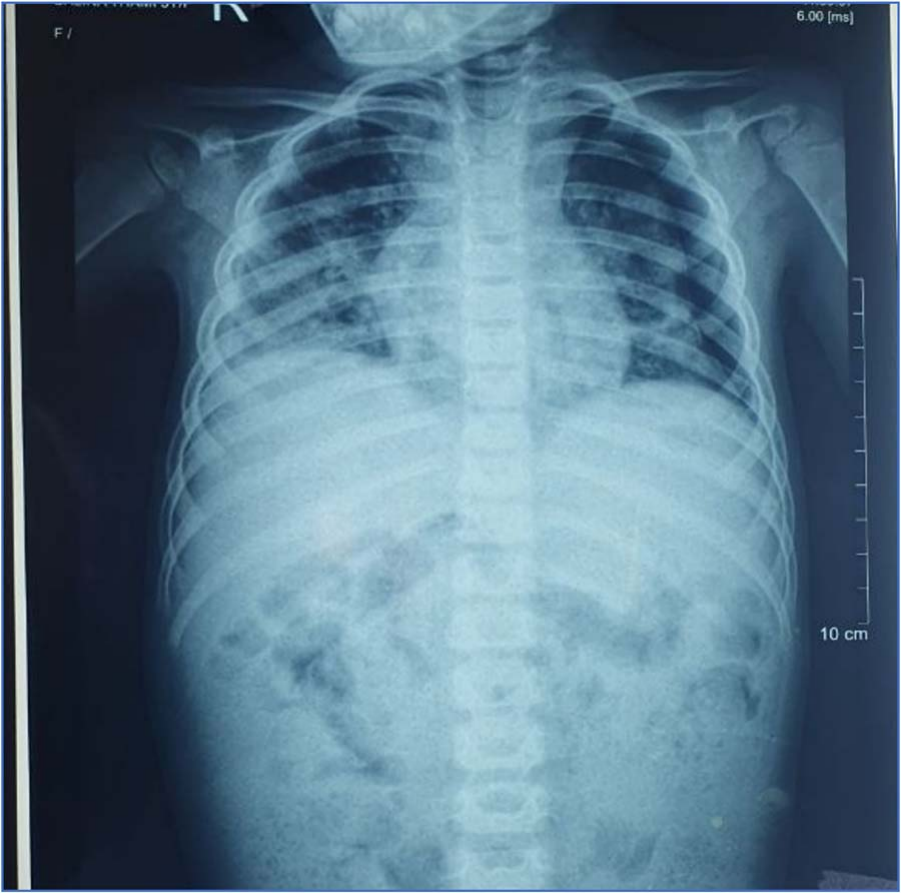
Chest x-ray anteroposterior view showing homogenous opacity with blunting of the costophrenic angle suggestive of pleural effusion.

**Figure 2:**
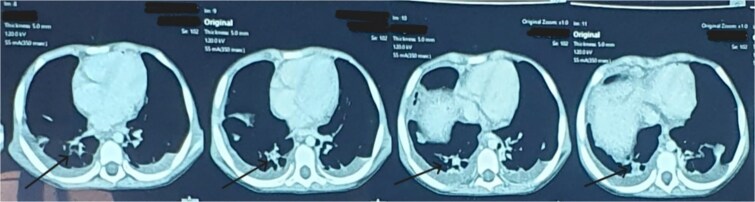
CT scan (axial view) showing moderate fluid collection in bilateral pleural cavities suggestive of bilateral pleural effusion (As shown by blue arrowheads).

**Figure 3:**
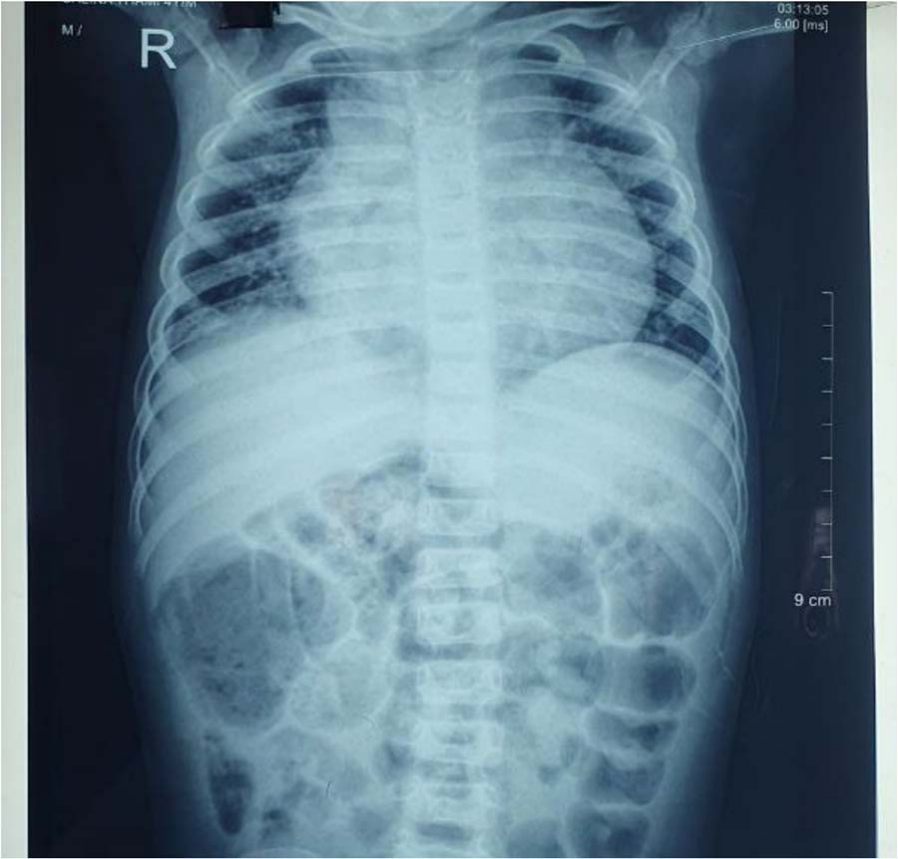
Chest X-ray showing globular enlargement of the cardiac shadow was seen, giving a water bottle appearance, suggesting pericardial effusion.

On a posteroanterior view of the chest radiograph, homogenous opacity with blunting of costophrenic angle suggestive of pleural effusion was seen (Fig. 1). A CT scan was also performed, confirming bilateral pleural effusion (Fig. 2). Upon repeating the chest x-ray after a few days, globular enlargement of the cardiac shadow was seen giving a water bottle appearance suggesting pericardial effusion. (Fig. 3) Echocardiography was done subsequently, which revealed moderate to large pericardial effusion with impending tamponade, and she was planned for pericardiocentesis, and 120 ml of straw-colored fluid was aspirated. Pericardial fluid ADA level and LDH were within normal limits. GeneXpert and AFB stain of pericardial fluid were performed, which came out negative. A rheumatological workup, including ANA, ANCA, and IgE levels, revealed normal results. Interestingly, the GeneXpert and Mantoux tests also came out normal. Bone marrow aspiration and biopsy were also performed, which revealed eosinophilic-rich cells without any atypical cells, ruling out any malignancy.

The patient was initially started on empirical anti-helminthic therapy. As stool microscopy was negative for ova and parasites, parasitic etiology was effectively ruled out. She was given prednisolone at 1 mg/kg/day for 1 week, following which the eosinophil count reduced, along with pericardiocentesis, which helped to alleviate the symptoms. She was discharged and advised to do an outpatient follow-up to monitor chest X-ray and eosinophil counts, which came out normal. Prednisolone was tapered over 1 month. The patient remained asymptomatic at a 3-month follow-up with a normal eosinophil count and normal follow-up imaging.

## Discussion

According to a 2011 working conference on eosinophil disorders and syndromes, the hypereosinophilic syndrome is defined when AEC is more than 1500/mm3 and evidence of end-organ dysfunction after excluding major diseases/conditions that may contribute to organ damage [4]. Common causes of Hypereosinophilia include allergic disorders, Helminthic infestations, drugs (allopurinol, carbamazepine, phenytoin, etc.), hematological disorders (such as leukemia, lymphoma, and myelodysplastic syndromes), and immunological disorders such as Vasculitis [7]. These conditions should be ruled out before coming to a diagnosis of Hyper-eosinophilic syndrome.

A patient with HES can have a wide range of complaints involving various systems, such as dermatological, pulmonary, cardiac, hematological, and gastrointestinal [7]. Clinical features also vary depending on the subtype of HES. In a study of 347 cases of HES by Requeena et al.,79% of the cases of lymphocytic HES present with skin manifestations, followed by abnormalities of bone marrow (41.9%) and lymph nodes (33.9%). In cases of myeloid HES, splenomegaly was most common (64.5%), followed by bone marrow abnormalities, heart, and liver impairment. In idiopathic HES, symptoms related to the heart, bone marrow, and lungs were common [8]. In patients with HES, pleural effusion is commonly reported, whereas pericardial effusion and ascites are rarely reported [9]. This case had pleural effusion, subsequently followed by pericardial effusion. Skin and lymph node manifestations were absent, and splenomegaly was also not present; findings were more consistent with idiopathic HES.

All patients with elevated Vitamin B12 and tryptase levels should undergo testing for BCR:ABL1, FIP1L1: PDGFRA mutations to rule out myeloproliferative HES. Also, T- and B-cell receptor rearrangement studies can be done to detect lymphocytic HES, but these tests could not be performed due to unavailability [10]. A thorough workup was done to find the cause of HES. CBC revealed hypereosinophilia, for which rheumatological, infectious, and neoplastic causes were ruled out via multiple investigations. Thus, secondary causes of hypereosinophilia were ruled out. Furthermore, normal Vitamin B12 and tryptase levels ruled out myeloproliferative HES. As no obvious cause for hypereosinophilia was found, a diagnosis of idiopathic HES was made. Steroids (prednisolone 0.5-1 mg/kg/day) are considered the first line of therapy for the treatment of hypereosinophilic syndrome [3]. Although steroids are effective in producing an initial response, toxicity and resistance may occur in the long term, limiting their use [10]. Antiparasitic and anticoagulant therapy should be combined with steroids in acute-onset HES. Hydroxyurea and Interferon alpha are used as second-line agents for idiopathic HES, but limited evidence supports their use [7]. Prednisolone was given after ruling out any possible underlying cause, which helped relieve the symptoms and decrease eosinophil counts.
